# A Rare Case of Pelvic Organ Prolapse in a Nulliparous Female With Autosomal Dominant Polycystic Kidney Disease (ADPKD) and Polycystic Liver Disease (PLD)

**DOI:** 10.7759/cureus.20023

**Published:** 2021-11-29

**Authors:** Sharon Hechter, Nagapratap Ganta, Smriti Kochhar, Anish Kanukuntla, Priyaranjan Kata, James Turro, Pramil Cheriyath

**Affiliations:** 1 Internal Medicine, Hackensack Meridian Health Ocean Medical Center, Brick, USA; 2 Medicine, Rowan School of Osteopathic Medicine, Stratford, USA; 3 Internal Medicine, Hackensack Meridian Ocean Medical Center, Brick, USA; 4 Internal Medicine, Hackensack Meridian Health, Ocean Medical Center, Brick, USA

**Keywords:** cyst, end-stage renal disease, pelvic organ prolapse, polycystic liver disease, autosomal-dominant polycystic kidney disease

## Abstract

Polycystic liver disease (PLD) is a condition that most often occurs in patients with autosomal dominant polycystic kidney disease (ADPKD) and less commonly as isolated liver disease. The presence of both conditions has proven to be a therapeutic challenge. Patients with ADPKD can suffer from significant renal and extra-renal complications and symptoms as a result of space-occupying cysts from polycystic kidney and liver enlargement. We present a case of ADPKD in a 56-year-old Caucasian female who developed pelvic organ prolapse, a rare complication, while also dealing with multiple other complications of ADPKD. Despite the high prevalence of ADPKD, complications such as pelvic organ prolapse have seldom been reported and discussed in the literature. The care team should do a prompt gynecological examination when they realize the burden of cysts becomes so large.

## Introduction

Autosomal-dominant polycystic kidney disease (ADPKD) is the most common inherited disease of kidneys due to mutations in the PKD1 gene located on chromosome 16p13.3, encoding polycystin-1, which is a large integral membrane protein that plays a role in adhesion and extracellular matrix regulation. ADPKD type I accounts for 85% to 90% of ADPKD cases [1]. ADPKD is characterized by a progressive increase of bilateral renal cysts, recurrent urinary tract infections, kidney stones, and hematuria which results in chronic kidney disease (CKD) and often leads to end-stage renal disease (ESRD). ADPKD comprises 5-10% cases with end-stage renal disease (ESRD) [1]. ADPKD affects multiple systems including hepatic, pancreatic, and arachidonic cysts, intracranial aneurysms, hypertension leading to cardiac complications, colonic diverticular disease, abdominal hernias, fertility issues, and bronchiectasis. However, pelvic organ prolapse is a rare complication that indirectly results from ADPKD. Various screening tests are recommended to ADPKD patients to diagnose these complications early and to prevent morbidity and mortality.

Polycystic liver disease (PLD) is a common extra-renal manifestation in patients with ADPKD. The prevalence of PLD in patients with ADPKD ranges from 20% to 75%. The pathogenesis of PLD associated with ADPKD involves cell adhesion disruption as the primary defect. The majority of patients that present with isolated PLD are usually asymptomatic. However, patients with liver cysts associated with ADPKD are more prone to symptoms related to renal failure and liver cysts account for up to 10% mortality in ADPKD patients on hemodialysis [2]. We report a rare complicated case of compression of the surrounding abdominal organs secondary to ADPKD and PLD in a 56-year-old Caucasian female. This case is unique in that she had an accompanying pelvic organ prolapse as a complication of enlarged liver and kidneys.

## Case presentation

A 56-year-old Caucasian nulliparous female presented to our outpatient clinic with left lower quadrant abdominal discomfort. The discomfort was described as a fluid wave that radiates from the left lower quadrant to the umbilicus, which was worse after eating. For the past 6 years, she had suffered from a 4/10 chronic flank pain. She has a history of ADPKD, multiple liver cysts along with other complications of ADPKD including stage 3 CKD, nephrolithiasis, constipation, mitral valve prolapse, pelvic organ prolapse. Ten years ago she had a total hysterectomy with bilateral salpingo-oophorectomy. Six months ago, she underwent robotic-assisted laparoscopic sacral colpopexy, paravaginal defect repair with cystocele repair, rectocele repair with perineorrhaphy using mesh. She has no children. Family history is significant with a father with polycystic kidney disease, hypertension, aneurysm, and heart disease, a mother with ovarian cancer. Her vital signs were unremarkable with weight 116 lb (52.6 kg), pulse 68 BPM, temperature 98.8℉ (37.1 ℃), blood pressure 108/62 mmHG, BMI of 20.34 (18.5-24.9) and hyposthenia body habitus. On the physical exam, she had a +3 systolic murmur with a mid-systolic click at the apex; the abdomen was diffusely distended with two-third of the abdomen filled with palpable masses from the liver and kidney cysts and tenderness to palpation.

**Figure 1:**
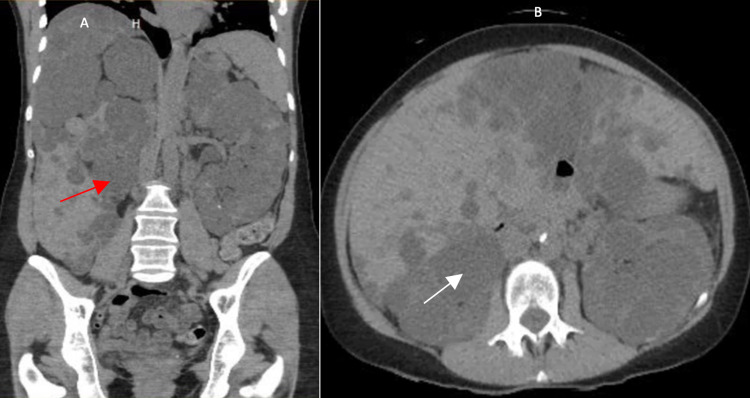
Non-contrast computed tomography coronal view (A) and axial view (B) of the abdomen showing simple and complex cysts within the parenchyma of the kidney (red arrow). Extensive cysts with coarse calcifications (white arrow) can be appreciated in bilateral kidneys.

**Figure 2:**
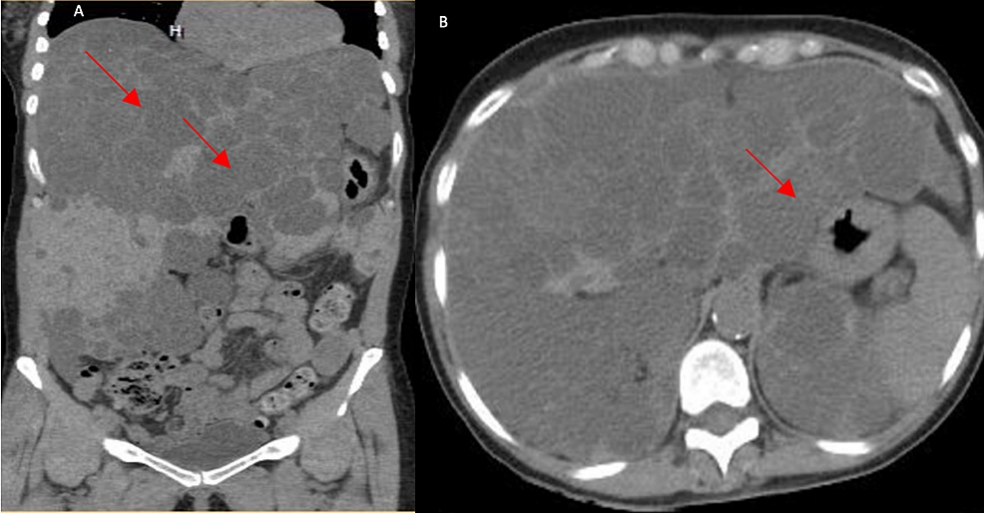
Non-contrast computed tomography coronal view (A) and axial view (B) of the abdomen showing hyperdense cysts within the parenchyma of the liver and a diffusely enlarged liver (red arrow).

Labs showed elevated blood urea nitrogen (BUN) of 51 mg/dl (7-18 mg/dL), creatinine of 2.14 mg/dL (0.6-1.2 mg/dL), decreased eGFR of 24ml/min (120−130 mL/min), and low sodium of 133 mEq/L (136-146 mEq/L). Complete blood count shows low RBCs of 3.94*10^6/µL (4.10-5.10*10^6/µL), and hemoglobin of 11.9 g/dL (12-16 g/dL). Renal ultrasound showed enumerable simple and complex cysts in bilateral kidneys, with kidneys measuring 13.7 x 7.6 x 7.2 cm on the left and 12.2 x 6.1 x 6.3 on the right. Abdominal ultrasound shows liver size measuring 20.4 in the cranial-caudal direction, with innumerable simple cysts seen throughout the liver. An echocardiogram showed mildly dilated left atrium with impaired diastolic dysfunction. Moderate to severe mitral valve prolapse and mitral regurgitation is also appreciated. Non-contrast Computed tomography (CT) scan of the abdomen and pelvis showed extensive cysts with coarse calcifications in bilateral kidneys (Figures 1A, 1B) and numerous simple cysts throughout the liver parenchyma (Figures 2A, 2B). Her blood pressure is medically controlled with 160 mg of amlodipine-valsartan and 2.5 mg of Nebivolol. She was also in 50 mg of tramadol as needed for pain for the past one year.

## Discussion

ADPKD is the most common inherited cause of kidney disease which accounts for 4.7% of end-stage renal disease in the United States [3].

We hypothesize space-occupying lesions are the main cause of cystocele and rectocele complications in patients with ADPKD. Space occupying lesions displace the surrounding organs to the periphery causing an increase in intra-abdominal pressure. Moreover, another cause of increased intra-abdominal pressure can be constipation, which is also caused by opioids that our patient was on for chronic pain. In a study by Kim et al., the pressure burden of space-occupying cysts caused complications such as leg edema (20.4%), ascites (16.6%), and hernia (3.6%) were common, the risk of such complications increased by sixfolds in patients with moderate to severe PLD [4]. As in this patient, at least two-thirds of the abdominal cavity is occupied by the liver and kidney cysts displacing and compressing the bowels and other soft tissue towards the pelvis, as seen in Figures 1A, 2A.

Adding to the burden of space-occupying lesions, hysterectomy increases the risk of pelvic floor prolapse as it disturbs the uterine ligamentous support [5]. The above patient did not have the support of a uterus and ligaments to dissipate the force from increased abdominal pressure resulting in prolapse of the bladder into the vaginal vault. Risk factors for pelvic organ prolapse include age, childbirth, estrogen deficiency, and chronically raised intra-abdominal pressure [6]. In our patient hysterectomy with bilateral oopherectomy was protective as estrogen caused an increase in liver cyst growth [6]. We suspect the hysterectomy, and comorbidities of cyst burden from both ADPKD and PLD could have significantly increased her risk of pelvic organ prolapse. 

Most of the patients are diagnosed with ADPKD through abdominal ultrasound either incidentally or as a part of family history. Routine gynecological examination is recommended in any woman with ADPKD especially with a sudden drop in eGFR to determine the risk of pelvic organ prolapse and to provide early intervention [7]. The drop in eGFR can be linked to obstruction in the urinary tract caused by the cystocele leading to hydronephrosis. As in our patient, she had pelvic organ prolapse at the same time while she progressed from stage 3 CKD to stage 4 CKD. A computed tomography (CT) scan of the abdomen without contrast is helpful in identifying the size, location, and extent of the cysts.

Control of blood pressure with antihypertensive agents especially by angiotensin-converting enzyme (ACE) inhibitors or angiotensin-receptor blockers (ARBs) has shown improved LVH and mortality in ADPKD patients. Chronic pain in these patients should not be treated with opioids as they can aggravate constipation, hence leading to increased abdominal pressure. Somatostatin analogs such as octreotide or lanreotide are shown to be beneficial for patients with PLD as they can help in both symptom relief and liver volume reduction [3]. Cyst fenestration with hepatic segmental resection can be considered in patients who are significantly incapacitated and suffer from severe symptoms as a result of the massive polycystic liver [8]. In patients with pelvic prolapse, pelvic floor repair and surgical correction of prolapse are recommended. Liver and kidney transplantation is the only definitive treatment for those with severe polycystic kidney and liver disease.

## Conclusions

Compression of surrounding organs is a well-known complication in patients with ADPKD and PLD. Pelvic organ prolapse is a rare complication in a nulliparous woman while underlying ADPKD could be the probable etiology. Despite the high prevalence of ADPKD, there are still a lot of unknown pathophysiology mechanisms and associations of many extra-renal manifestations. Nevertheless, nephrologists and physicians should be aware of the possibility of such rare complications and manage accordingly in a timely manner to prevent morbidity and mortality.
